# Abacavir Induced T Cell Reactivity from Drug Naïve Individuals Shares Features of Allo-Immune Responses

**DOI:** 10.1371/journal.pone.0095339

**Published:** 2014-04-21

**Authors:** Jacqueline Adam, Natascha Wuillemin, Stephan Watkins, Heidi Jamin, Klara K. Eriksson, Peter Villiger, Stefano Fontana, Werner J. Pichler, Daniel Yerly

**Affiliations:** 1 Clinic for Rheumatology and Clinical Immunology/Allergology, University Hospital of Bern, Bern, Switzerland; 2 Regional Blood Transfusion Service of the Swiss Red Cross, Bern, Switzerland; New York University, United States of America

## Abstract

Abacavir hypersensitivity is a severe hypersensitivity reaction which occurs exclusively in carriers of the HLA-B*57∶01 allele. *In vitro* culture of PBMC with abacavir results in the outgrowth of abacavir-reacting CD8^+^ T cells, which release IFNγ and are cytotoxic. How this immune response is induced and what is recognized by these T cells is still a matter of debate. We analyzed the conditions required to develop an abacavir-dependent T cell response *in vitro*. The abacavir reactivity was independent of co-stimulatory signals, as neither DC maturation nor release of inflammatory cytokines were observed upon abacavir exposure. Abacavir induced T cells arose in the absence of professional APC and stemmed from naïve and memory compartments. These features are reminiscent of allo-reactivity. Screening for allo-reactivity revealed that about 5% of generated T cell clones (n = 136) from three donors were allo-reactive exclusively to the related HLA-B*58∶01. The addition of peptides which can bind to the HLA-B*57∶01-abacavir complex and to HLA-B*58∶01 during the induction phase increased the proportion of HLA-B*58∶01 allo-reactive T cell clones from 5% to 42%. In conclusion, abacavir can alter the HLA-B*57∶01-peptide complex in a way that mimics an allo-allele (‘altered self-allele’) and create the potential for robust T cell responses.

## Introduction

Delayed type hypersensitivity reactions (HR) to drugs are mediated by the adaptive immune system. How such small molecules are able to induce an immune response is not understood yet. Drug HR involve the generation of drug-reacting T cells, some of which occur preferentially or exclusively in individuals carrying defined human leukocyte antigen (HLA)-class I (and rarely class II) alleles [1]. One of the best investigated examples of these HLA allele associated drug HR is hypersensitivity to abacavir, a competitive inhibitor of the HIV reverse transcriptase [2]. All of the patients who develop severe abacavir HR carry the HLA-B*57∶01 allele, whereas 47.9% of the HLA-B*57∶01^+^ individuals would develop HR upon abacavir exposure. Therefore, screening for HLA-B*57∶01 has become standard before starting anti-HIV therapy with abacavir, which represents a very successful example of personalized medicine.

The stimulation of T cells via the T cell receptor (TCR) can be explained by different mechanisms which can also occur together. The hapten theory assumes that drugs covalently modify macromolecules, leading to the presentation of haptenized peptides to T cells. In many contact hypersensitivity models, immune responses to haptens have been investigated and revealed a complete immune response (reviewed in [3]). Indeed, the hapten formation by chemically highly reactive substances like halogenated phenol derivatives leads first to the maturation of DC, which then prime naïve T cells against haptenized peptides. Nevertheless this model cannot explain most of drug HR, because most drugs do not undergo covalent bindings to proteins. Therefore, the p-i (pharmacological interaction with immune receptor) concept has been developed, postulating that drugs or metabolites bind by van der Waals and electrostatic interactions to immune receptors like they do to other proteins [4]. In the case of carbamazepine-reacting T cells for instance, the drug makes interactions between defined TCR clonotypes and the HLA-B*15∶02 molecule [5]. When these drug-immune receptor interactions have sufficient affinity, they may elicit an immune response. Consequently, drug binding to crucial positions of the complementary determining regions on the TCR has been defined as ‘p-i TCR’, whereas preferential binding to HLA molecules have been referred to ‘p-i HLA’ (reviewed in [6]).

Recent studies have shown that abacavir binds non-covalently within the F-pocket of the HLA-B*57∶01 peptide binding groove [7]–[9]. When provided in very high concentrations (100 µg/ml), it can modify the peptide binding properties of HLA-B*57∶01. In this case, peptides with a small aliphatic residue at the C-term are favored in comparison to peptides with Trp or a Phe, which are normally found on unmodified HLA-B*57∶01 molecules. How the altered HLA-abacavir-peptide complex is actually stimulating T cells is not yet clear. It has been speculated that an autoimmune or an allo-immune like reaction to this altered peptide-HLA complex could take place. However, neither specificity of abacavir-reacting T cells nor their possible peptide specificity have been proven yet. Moreover, we have previously shown by calcium influx measurements that the addition of abacavir to T cell clones (TCC) elicits an immediate reactivity in a fraction of them [10]. This makes a peptide exchange less plausible, since peptide loading is supposed to occur in the endoplasmatic reticulum (ER) and to require more time. Since all these abacavir reacting TCC were generated from abacavir naïve individuals, the relevance of such cells in the pathogenesis of abacavir HR remains to be confirmed.

In this study we investigated how the abacavir-HLA complex {HLA-B*57∶01+abc} initiates an immune response. We showed *in vitro* that the {HLA-B*57∶01+abc} complex stimulated T cell expansion in a DC independent manner. The abacavir-reacting T cells derived from naïve and memory T cell pools. This type of T cell activation by abacavir resembled an allo-immune stimulation. Besides, abacavir-reacting TCC cross-reacting exclusively with HLA-B*58∶01 molecules were found in TCC generated from three individuals. Finally, the addition of peptides naturally fitting into the HLA-B*58∶01 peptide binding groove and into the {HLA-B*57∶01+abc} complex enhanced the strength and the frequency of allo-reactive-, abacavir-reacting T cells. Taken together, we concluded that abacavir hypersensitivity shows features related to an allo-immune response *in vitro*.

## Materials and Methods

### Healthy Donors and HIV^+^ Abacavir Naïve Patients

Thirteen abacavir naïve HLA-B*57∶01^+^ healthy donors (HD) were selected from Bern’s blood donation center according to their HLA-B*57∶01 status and enrolled in the study. Furthermore, 7 HIV-positive, HLA-B*57∶01^+^ individuals who were never exposed to abacavir were enrolled in the study.

### Primary Stimulation, TCL and TCC Generation

PBMC were isolated by Ficoll density gradient centrifugation and cultured in RPMI-1640 (Gibco, Basel, Switzerland) supplemented with 10% of heat-inactivated human AB serum (Swiss Red Cross, Bern, Switzerland), 2 mM L-Glutamine (Biochrom, Berlin, Germany), 25 µg/ml transferrin (Biotest, Dreieich, Switzerland), 50 U/ml penicillin and 50 µg/ml streptomycin (Bioconcept, Allschwil, Switzerland). Abacavir-reacting T cells were enriched by culturing lymphocytes (4×10^6^ cells in 2 ml) in 10 µg/ml abacavir-sulfate (Reseachem, Burgdorf, Switzerland). If mentioned, selected peptides (see below) were added concomitantly with abacavir at 10 µg/ml. Cells were supplemented with 50 IU/ml IL-2 (Roche, Basel, Switzerland) every other day starting from day 5 to day 14 of T cell culture. Abacavir-reacting TCL were further expanded for three rounds of restimulations in the presence of irradiated autologous PBMC and 10 µg/ml abacavir in solution. Furthermore, primary induction was also performed with 14 days old phytohaemaglutinine (PHA) -blasts and negatively selected CD3^+^ T cells, using magnetic sorting (Stemcell Technologies, Grenoble, France) according to manufacturers’ instruction.

Cloning was performed by limiting dilution as described previously [11]. Specificity testing was performed by IFNγ ELISpot and cytotoxicity assays as described elsewhere [10]. TCC were considered abacavir-reacting if cytotoxicity in the culture exceeded >30% and/or when the difference in the number of spot forming cells between wells with and without abacavir exceeded 50. Clones were selected for functional studies based on antigen specificity and the availability of cells. Clones that were resistant to expansion were not used in functional studies.

### Enrichment for Naïve or Memory CD8^+^ T Cells

Human naïve or memory CD8^+^ T cells were isolated from PBMC with EasySep negative selection kits (Stemcell Technologies, Grenoble, France) for the enrichment of human naïve CD8^+^ T cells or human memory CD8^+^ T cells, respectively, according to the manufacturer’s protocols. Briefly, freshly isolated PBMC were incubated either with naïve CD8^+^ T cell enrichment cocktail and CD45RO depletion cocktail (for naïve enrichment) or with memory CD8^+^ T cell enrichment cocktail (for memory enrichment) and EasySep magnetic particles. Magnetically labelled cells were separated from unlabelled cells with the EasySep Magnet. The negatively selected enriched cells were washed in PBS and induced with 10 µg/ml abacavir as described above.

### Reactivity Testing Upon Abacavir Stimulation

On day 7 to 14 after primary stimulation, reactivity of TCL was examined by flow cytometry. Cells were washed three times and incubated in the presence or absence of 10 µg/ml abacavir for 6 h with CFSE labelled autologous PBMC. After 2 h of co-incubation Brefeldin A (10 µg/ml, Sigma-Chemicals, Buchs, Switzerland), Monensin (6 µg/ml, Sigma-Chemicals, Buchs, Switzerland) and anti-CD107a-PE antibody (Biolegend, San Diego, CA, USA) were added. Surface staining was performed with anti-CD3-PerCp-Cy5.5, anti-CD4-PE-Cy7 and anti-CD8-APC-Cy7 (Biolegend, San Diego, California). Intracellular staining with anti-IFNγ-APC was performed according to the Cytofix/Cytoperm permeabilization kit (BD Biosciences, Basel, Switzerland). FACS analysis was performed on a FACSCanto-I cytometer using FACS-Diva software (BD Biosciences).

### In vitro Generation and Maturation of DC

CD14^+^ cells were enriched from freshly isolated PBMC with human CD14 MicroBeads (Miltenyi Biotec, Bergisch Gladbach, Germany) and MS separation columns (Miltenyi Biotec, Bergisch Gladbach, Germany) according to the manufacturer’s protocol. DC were generated by cultivation of CD14^+^ cells with 800 IU/ml GM-CSF (PeproTech EC Ltd, London, United Kingdom) and 1000 IU/ml IL-4 (Miltenyi Biotec, Bergisch Gladbach, Germany) for 5 days. After 5 days, DC were incubated with increasing concentrations of abacavir (3, 10, 30 µg/ml) or NiSO4 (250 mM). After 24 h of co-incubation, DC were harvested, and the expression of co-stimulatory molecules (CD80, CD83, CD86 and CD40 (Anti-bodies from BD Biosciences, Basel, Switzerland)) was analyzed by flow cytometry. Furthermore, culture supernatants were analyzed for IL-1β, TNFα and IL-6 cytokine secretion by multiplex analysis (Meso Scal Discovery, Gaithersburg, MD USA).

### Testing for Allo-reactivity of Abacavir-reacting TCC

Abacavir-reacting TCC were tested for allo-reactivity against a panel of EBV-transformed B-lymphoblastoid cell lines (EBV-BLCL, table 1) in a 16 h IFNγ ELISpot assay as described above. Briefly, abacavir-TCC were incubated with distinct APC at a ratio of 2,500 TCC per 40,000 APC on 96 well filtration plates (Millipore, Volketswil, Switzerland) coated with anti human IFNγ antibody. ELISpot plates were developed and spots were analyzed in a Bioreader 3000 CL/PrO (BIO-SYS GmbH, Karben, Germany) and IFNγ secretion was measured in terms of spot forming units (SFU). For confirmation of the cross-reacting HLA molecules, TCC were stimulated with the EBV transformed human lymphoid cell line 721.221 (.221) expressing a single HLA class I molecule of interest (HLA-B*57∶01 or HLA-B*58∶01) and analyzed by flow cytometry. 221 transfectants were generated according to Adam et al. [10].

**Table 1:** HLA typing of healthy donors included for allo-reactivity screening.

#	Donor	HLA-A		HLA-B		HLA-C	
1	ID-11	A*02	A*26	B*44	B*60	C*03	C*05
2	ID-674	A*01	A*11	B*35	B*57	C*04	C*06
3	ID-121	A*24	A*31	B*63	B*63	nd	nd
4	ID-32	A*01	A*02	B*08	B*51	C*07	C*15
5	ID-135	A*02	A*02	B*13	B*44	nd	nd
6	ID-274	A*02	A*31	B*13	B*18	nd	nd
7	ID-24	A*03	A*24	B*27	B*50	nd	nd
8	ID-189	A*03	A*30	B*57	B*27	nd	nd
9	ID-535	A*01	A*24	B*44	B*51	nd	nd
10	ID-60	A*26	A*28	B*51	B*51	nd	nd
11	ID-140	A*02	A*11	B*27	B*35	nd	nd
12	ID-492	A*31	A*32	B*40	B*51	C*02	C*05
13	ID-602	A*01	A*02	B*08	B*57∶01	C*06	C*07
14	ID-542	A*01	A*24	B*08	B*57∶01	C*06	C*07
15	ID-585	A*02	A*03	B*07	B*57∶01	C*06	C*07
16	ID-576	A*01	A*02	B*37	B*57∶01	C*06	C*07
17	ID-587	A*01	A*24	B*15	B*57∶01	C*03	C*06
18	ID-601	A*01	A*32	B*08	B*58∶01	C*03	C*07
19	ID-618	A*02	A*80	B*07	B*57∶01	C*06	C*07
20	ID-617	A*02	A*31	B*07	B*57∶01	C*06	C*07
21	ID-676	A*01	A*24	B*08	B*35	C*04	C*07
22	ID-164	A*01	A*30	B*18	B*27	nd	nd

Alleles from all HLA supertype families except B62 were included (A1, A2, A3, A24, B7, B8, B27, B44, B58).

### Selection of HLA-B*58∶01 and HLA-B*57∶01 Binding Peptides

The lists of peptides eluted from HLA-B*57∶01 in the absence and presence of abacavir published in [8] were compared and only peptides found in the presence of abacavir were considerd for further analysis. The affinity for HLA-B*57∶01 and HLA-B*58∶01 was calculated for each peptide using the NetMHCpan server [12]. Five peptides showing the highest difference in binding affinity between HLA-B*58∶01 and HLA-B*57∶01 were selected (table 2) and synthetized (90% purity, ProImmune, UK). They all stem from endogenously produced proteins. The selection process is described in detail in the results section.

**Table 2:** Peptide candidates possibly involved in HLA-B*58∶01 allogenicity.

#	Sequence§	Length	Name†	HLA-B*57∶01 IC50(nM)	HLA-B*58∶01 IC50(nM)	Ratio‡
1	QANSHKERGW§	10	QW9	12226.6	286.1	0.0234
2	KAERERGITI	10	KI10	6034.9	232.1	0.0385
3	KSRGSGEQDW§	10	KW10	5057.6	432.1	0.0854
4	SSAKIVPKI	9	SI9	5419.1	506.1	0.0934
5	RSVALAVLA	9	RA9	5806.4	552.1	0.0951
6	QSAKTQIKL	9	QL9	6553.2	966.7	0.1475
7	STTRVKPFI	9	SI9	5572.9	849.1	0.1524
8	RSRDDADRRY§	10	RY10	5227.0	882.1	0.1688
9	KSRDGERTVY§	10	KY10	5311.8	973.5	0.1833

Peptide sequences were selected from the publication of Norcross et al, 2012 [8]. Peptides were considered only if they were eluted from cells treated with abacavir and were absent from abacavir untreated cells. IC50 values for HLA-B*57∶01 and HLA-B*58∶01 were calculated using the netMHC pan server [12].

§ Peptide with Trp, Phe or Tyr at their C-term were excluded from the candidate list.

† Name was given arbitrary with the letter of the first and the last residue of the peptide, followed by the number of amino acids.

‡ The ratio value corresponds to the IC50 for HLA-B*58∶01 divided by IC50 for HLA-B*57∶01. It may point to a difference of peptide affinity to both allotypes.

### Modelling of HLA-B*58∶01 with SI9 Peptide and {HLA-B*57∶01+abc} with SI9 Peptide

The model of HLA-B*58∶01 was generated using the PDB id 2RFX as a template and swiss-modeller [13]. A linear model of the peptide SI9 (SSAKIVPKI) and abacavir was generated in PyMol (The PyMOL Molecular Graphics System, Version 1.5.0.4 Schrödinger, LLC.). Both HLA-B*57∶01 and HLA-B*58∶01 were aligned and the peptide from 2RFX used to orient the generated peptide. Abacavir was aligned from docking, or by alignment with the x-ray structure of 3VRI, and a topology file created using topology tools for the small molecule. Each model was then energy minimized, and solvent added as SPC water and 0.14 M NaCl, 0.08 M KCl, 0.06 M MgCl_2_ and 0.04 M CaCl_2_ to a box of 90×90×90 nanometers. These were then further energy minimized, and allowed to equilibrate at room temperature (300 kelvin) for 300 picoseconds.

### Statistical Analysis

Statistical analyses were performed using GraphPad Prism5 (GraphPad Software, San Diego, CA, USA). Results are expressed as mean ± SD. Comparisons were drawn using unpaired t test or Mann–Whitney U test. Each experiment was at least repeated twice. p values were considered statistically significant if *p<0.05 (95% confidence interval).

### Study Approval

The local ethical committee (Kantonale Ethikkommission - Bern) assigned the number 206/08 to the study ‘Interaktionen von Medikamenten mit Antigen-spezifischen Rezeptoren auf T Zellen’ and gave formal approval of this study. All individuals gave written informed consent prior to being enrolled in the study.

## Results

### Abacavir-reacting Cells are Detected in 100% of HLA-B*57∶01^+^ Drug Naïve Individuals after 12–14 Days of in vitro Stimulation

**Figure 1:**
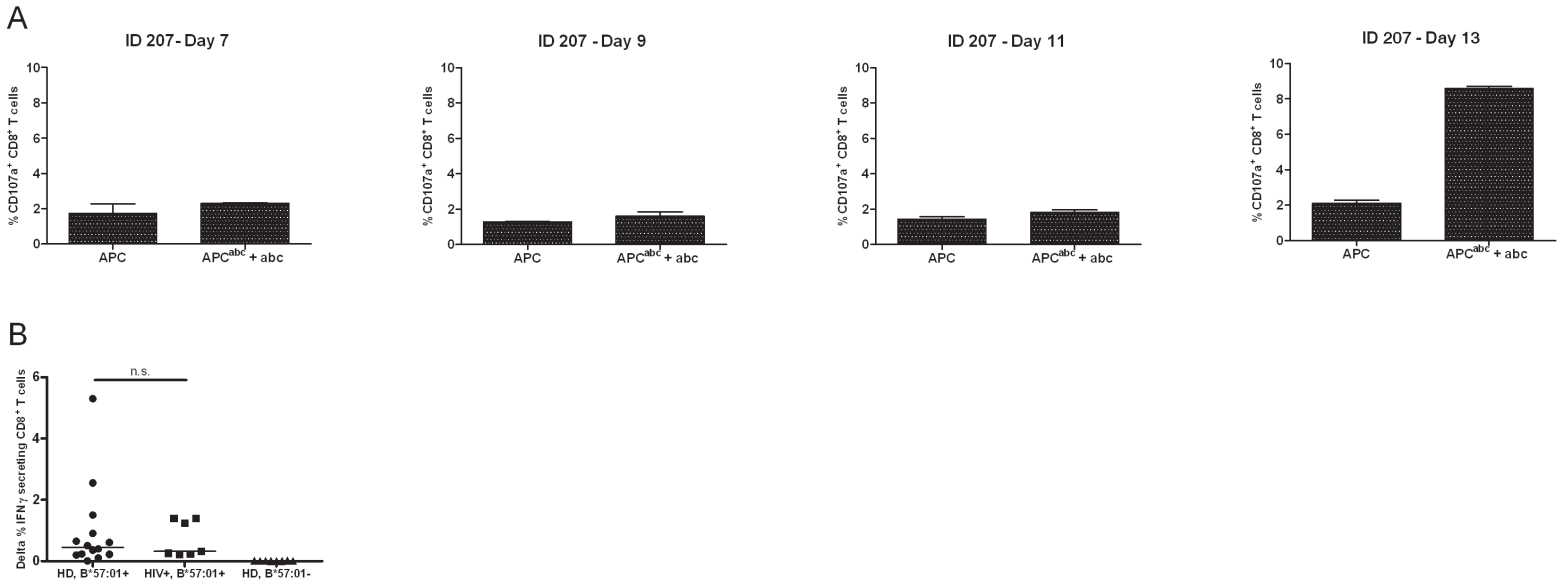
Abacavir-reacting CD8^+^ T cells are detectable after 13 days of *in vitro* culture and are observed in 100% of the tested HLA-B*57∶01^+^ individuals. A. PBMC from healthy donors (HD) were cultured *in vitro* with abacavir (10 µg/ml) for 14 days as explained in materials and methods. Reactivity was monitored after a drug-specific *in vitro* restimulation assay by flow cytometry. CD107a served as marker for T cell reactivity. Representative data from ID-207 are shown as mean ± SD. Experiment was performed in duplicates. B. PBMC from HLA-B*57∶01^+^ HD (n = 13), HLA-B*57∶01^−^ HD (n = 8) and HLA-B*57∶01^+^ HIV^+^ patients (n = 7) were induced with abacavir (10 µg/ml) for 14 days *in vitro*. T cell reactivity was monitored by means of IFNγ secretion after a drug-specific restimulation. p = 1.00, two tailed Mann-Whitney test.

We and others were able to induce a T cell response to abacavir after an *in vitro* stimulation of 14 days. This reactivity was never detected *ex vivo* in drug naïve individuals [14] Therefore, we investigated how long it actually takes for such a response to be detectable *in vitro*. PBMC from HLA-B*57∶01^+^ individuals were cultured in the presence of abacavir as described in ‘material and methods’. The reactivity of TCL was assessed by intracellular IFNγ expression and degranulation (Fig. S1) on FACS after 7, 9, 11 and 13 days (Fig. 1a) after re-challenge stimulation. Abacavir reactivity was detected only after 13 days in a sustainable and reproducible way. The addition of pro-inflammatory molecules like poly IC or LPS neither shortened the expansion time, nor increased the fraction of reacting CD8^+^ T cells (data not shown). Drug-reacting CD8^+^ T cells were observed in 100% of the 20 individuals included in the study (Fig. 1b), independent of their HIV status.

### The Expansion of Abacavir-reacting Cells Occurs Independently from the Innate Immune System

**Figure 2:**
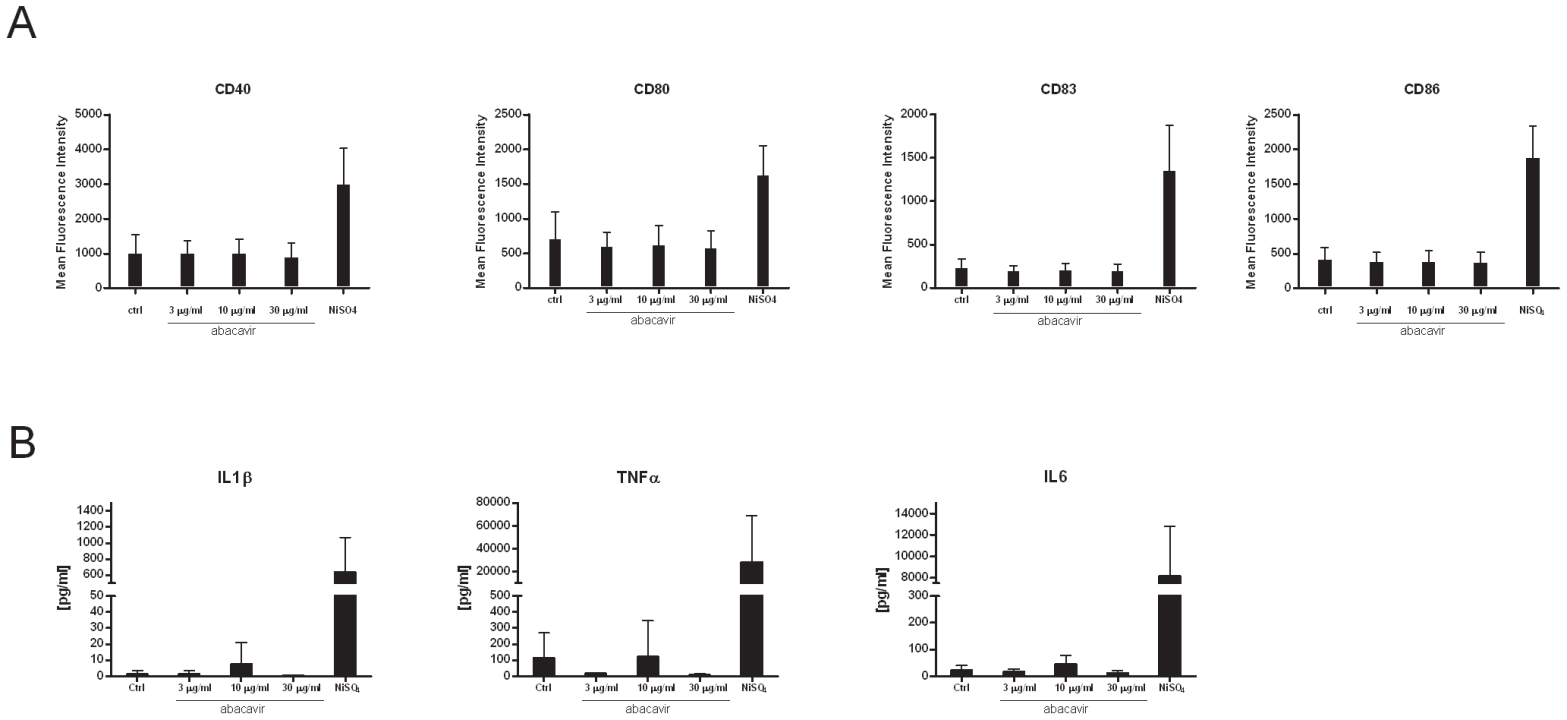
Abacavir does not induce DC maturation. *In vitro* generated DC were incubated with increasing concentrations of abacavir or NiSO_4_ (250 mM) for 24 hours. A. DC were harvested and the expression of co-stimulatory molecules was analyzed by flow cytometry. Data are shown as mean fluorescence intensity and represent mean ± S.E.M. from DC of 4 individuals. Experiments were performed in triplicates. B. Cell culture supernatants were analyzed for cytokines by multiplex analysis. Data represent mean ± S.E.M. from DC of 4 individuals. Experiments were performed in triplicates.

Since abacavir reactivity cannot be observed *ex vivo* and since 2 weeks are necessary to generate the immune response, we investigated whether abacavir was able to interact with the innate immune system to provide a danger signal. Therefore the effects of abacavir on DC were considered. Increasing concentrations of abacavir were added to *in vitro* generated myeloid DC. The expression of co-stimulatory molecules like CD80, CD86 and other co-activation markers such as CD40 and CD83 was evaluated by flow cytometry (Fig. 2a). The addition of nickel sulphate served as positive control for DC maturation. Up-regulation of maturation markers was never observed even with drug doses exceeding 3 times the concentration used to induce reacting T cells *in vitro* (30 µg/ml). Along with the evaluation of co-stimulatory markers, the culture supernatants were also evaluated for inflammatory cytokines (IL-1β, IL-6, TNFα). No activation or release of inflammation mediators was observed after the addition of abacavir (Fig. 2b). Altogether these results suggest that abacavir had no direct effect on DC.

**Figure 3:**
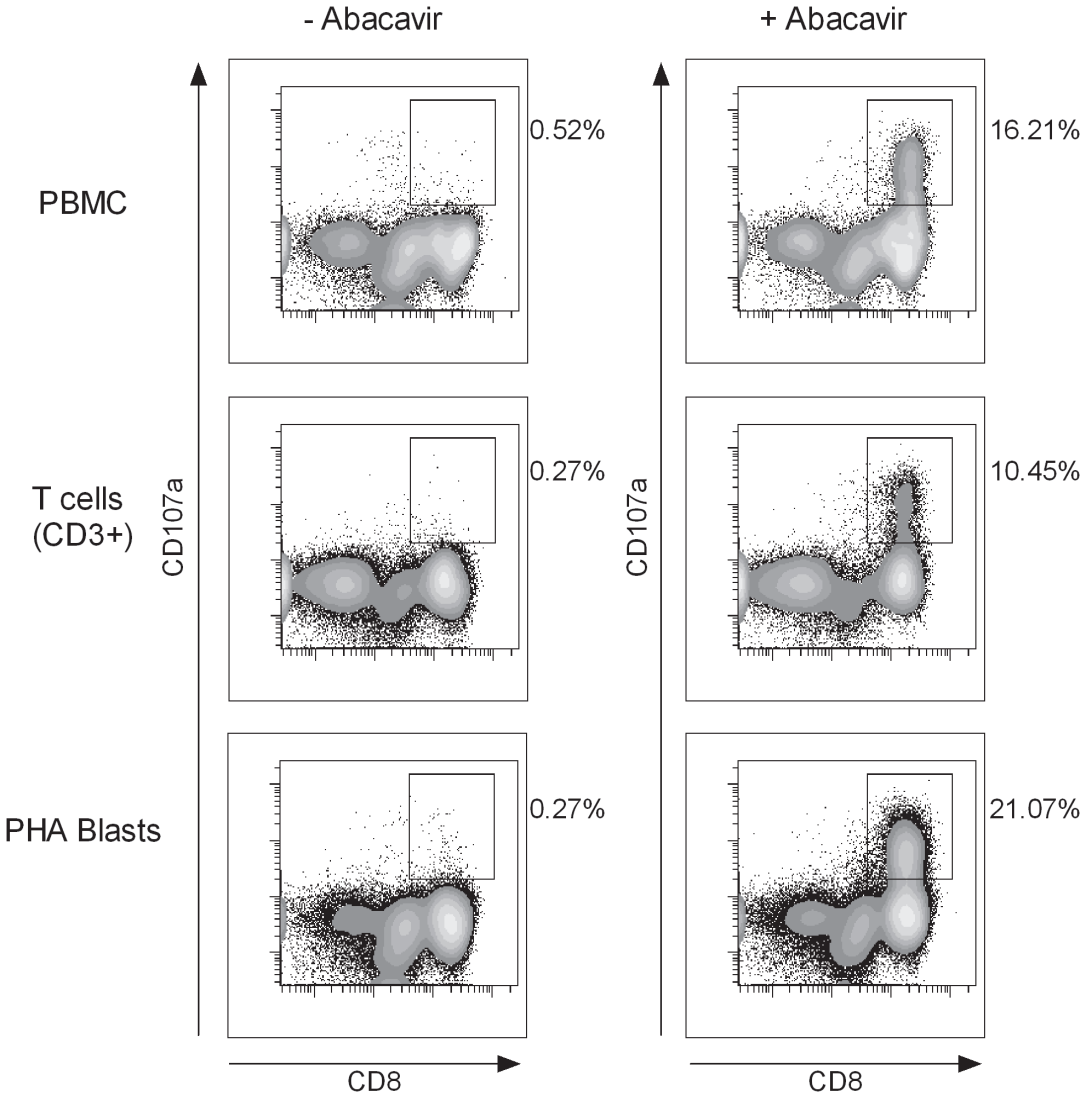
Abacavir-reacting T cells can be induced *in vitro* in the absence of APC. PBMC, CD3^+^ sorted T cells, and 14 days old PHA-blast from ID-453 were cultured in the presence of abacavir (10 µg/ml). Drug reactivity was monitored on day 14 in a degranulation (CD107a) assay after a six hours drug-specific restimulation assay. Plots are gated on CD3^+^ T cells and percentages relate to the CD8^+^ CD107a^+^ T cell population. Representative data from three independent experiments are shown.

Since abacavir did not activate DC to provide any danger signal, we wondered whether it was possible to induce abacavir reactivity in the absence of professional APC, i.e. in pure T cells cultures. For that purpose T cells (CD3^+^) were purified by negative selection using magnetic sorting. These purified T cells were stimulated with abacavir similarly to PBMC. Alternatively, T cells were first stimulated with PHA for 14 days and then specifically stimulated with abacavir for another 14 days. After abacavir re-challenge we observed sustained abacavir reactivity in all investigated fractions (Fig. 3). The frequency of abacavir-reacting T cells was higher when T cells were previously expanded with PHA. Therefore we concluded that primary immune responses to abacavir can take place even in the absence of professional APC.

### Abacavir-reacting Cells arise from the Naïve and Memory Cell Compartment

**Figure 4:**
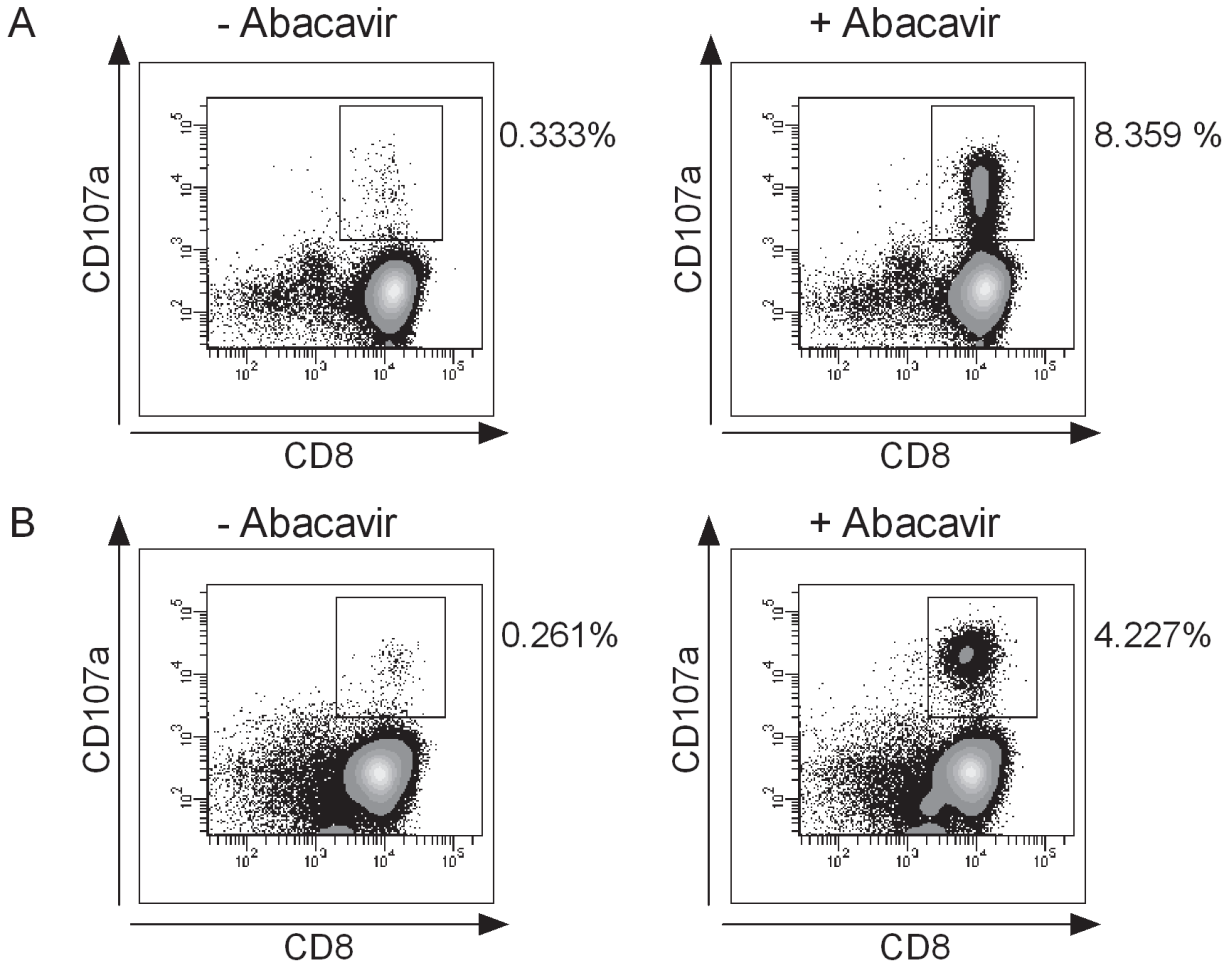
Abacavir-reacting T cells are found in the naïve and memory CD8^+^ T cell pools. CD8^+^ T cells from two HLA-B*57∶01^+^ donors were isolated by magnetic sorting according to their expression of memory (CD45RO) and naïve (CD45RA, CCR7) markers. (A) Negatively selected memory and (B) naïve CD8^+^ T cells were stimulated with abacavir (10 µg/ml). After 14 days, cells were re-challenged with abacavir (10 µg/ml) and reactivity was monitored by flow cytometry. Plots show CD3^+^ T cells and percentages relate to the CD8^+^ CD107a^+^ T cell population. Representative data from two independent experiments of ID-576 are shown.

Since abacavir reactivity could not be detected *ex vivo* and since it took 12–14 days for the induction of a robust immune response, we hypothesized that the abacavir-reacting cells arose from the naïve compartment. To assess this hypothesis, naïve and memory CD8^+^ T cells fractions were isolated by negative selection using magnetic sorting technology. The sorting purity was analyzed by flow cytometry (Fig. S2). Although purity was slightly below 90%, we considered it sufficient to pursue our experiments with these sorted fractions. Purified CD8^+^ T cells were stimulated with abacavir for 14 days (Fig. 4). Re-challenge with abacavir revealed abacavir reactivity in both T cell pools. Considering the amount of abacavir-reacting T cells, we can exclude that these cells arose from naïve cells contaminating the memory pool and vice versa. Of note, the induction of abacavir-specific immune response is not dependent on DC, because the investigated T cell fractions were cultured in the absence of any APC.

### Five Percent of Abacavir-reacting T Cell Clones are Allo-reactive to the HLA-B*58∶01 Molecule

**Figure 5:**
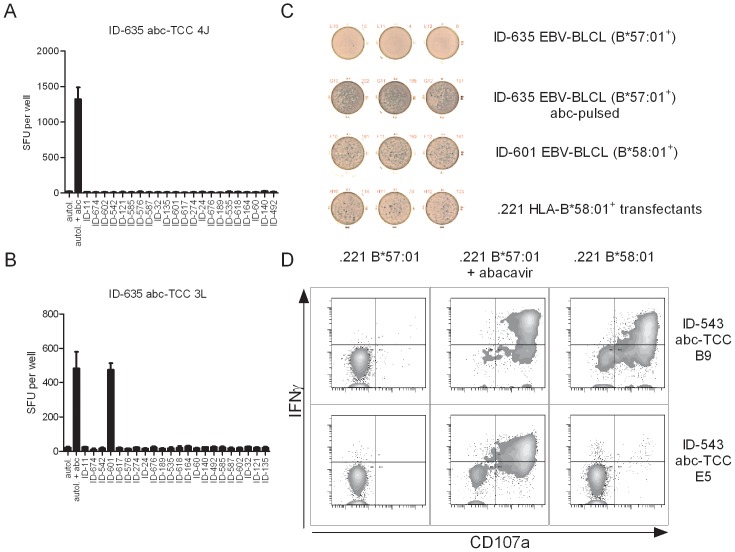
Allo-reactivity of abacavir-reacting T cell clones. Abacavir-reacting TCC from three individuals were tested for allo-reactivity against a panel of EBV-BLCL from 22 HD (table 1) in a 16 hour IFNγ ELISpot assay. A. An example of a TCC from ID-635 without allo-reactivity is shown. The experiment was performed in triplicates and data are shown as mean ± SD. B. TCC 3L from ID-635 shows allo-reactivity against EBV-BLCL from donor ID-601. The experiment was performed in triplicates and data are shown as mean ± SD C. Allo-reactivity of TCC ID-635 3L was confirmed by IFNγ ELISpot. TCC was stimulated with autologous EBV-BLCL (first line), abacavir-pulsed autologous EBV-BLCL (second line), EBV-BLCL from donor ID-601 (third line) and 721.221 cells expressing HLA-B*58∶01 (forth line). (D) TCC allo-reactive against HLA-B*58∶01 (upper panel) or without allo-reactivity to HLA-B*58∶01 (lower panel) were stimulated with 721.221 cells expressing HLA-B*57∶01 in the absence (left) or in the presence of abacavir (middle), or with 721.221 cells expressing HLA-B*58∶01 (right). After four hours of stimulation TCC were analyzed for CD107a up-regulation and IFNγ secretion by flow cytometry. Plots show gated CD3^+^ CD8^+^ T cells. Representative data of three independent experiments are shown.

The absence of co-stimulation and the occurrence of reacting cells from both pools (naïve and memory) are typical features of an allo-immune response [15]–[17] with high potency and elevated frequency of allo-reactive T cells [18]. The immunogenic determinant can either be the allogeneic HLA molecule, or the presented peptide or both [16]. Since our data show that abacavir-reacting T cells share several features with allo-reactive T cells, allo-reactivity or more particularly allo-specificity of abacavir-reacting T cells was evaluated. Abacavir-reacting TCC were generated from three individuals and their allo-specificity was assessed in an IFNγ ELISpot assay (Fig. 5 a–c). EBV-BLCL from a panel of 22 HLA mismatched donors, covering at least 34 different MHC-class I alleles representing 9/10 listed HLA superfamilies (table 1), were used as target cells. Seven out of the 136 screened abacavir-reacting TCC exhibited allo-reactivity against the same donor, i.e. HD-601 (table 3). Identification of the causative allele was done by flow cytometry and IFNγ ELISpot, using single HLA expressing 721.221 cells (Fig. 5 d). Transfectants expressing HLA-B*58∶01 stimulated allo-reactive TCC. Furthermore, clones were also activated in the presence of PBMC or PHA blasts from HLA-B*58∶01^+^ donors (Fig. S3).

**Table 3:** Summary of HLA-B*58∶01 allo-reacting TCC from abacavir-reacting CD8^+^ TCC.

Donor	abacavir-reacting TCC	HLA-B*58∶01 allo-reactive TCC
ID-453	n = 80	4 (5%)
ID-635	n = 21	1 (4.8%)
ID-618	n = 35	2 (5.7%)
TOTAL	N = 136	7 (5.1%)

### Peptides Presented on HLA-B*58∶01 and {HLA-B*57∶01+abc} Complex Increase the Frequency of Allo-reactive T Cells

**Figure 6:**
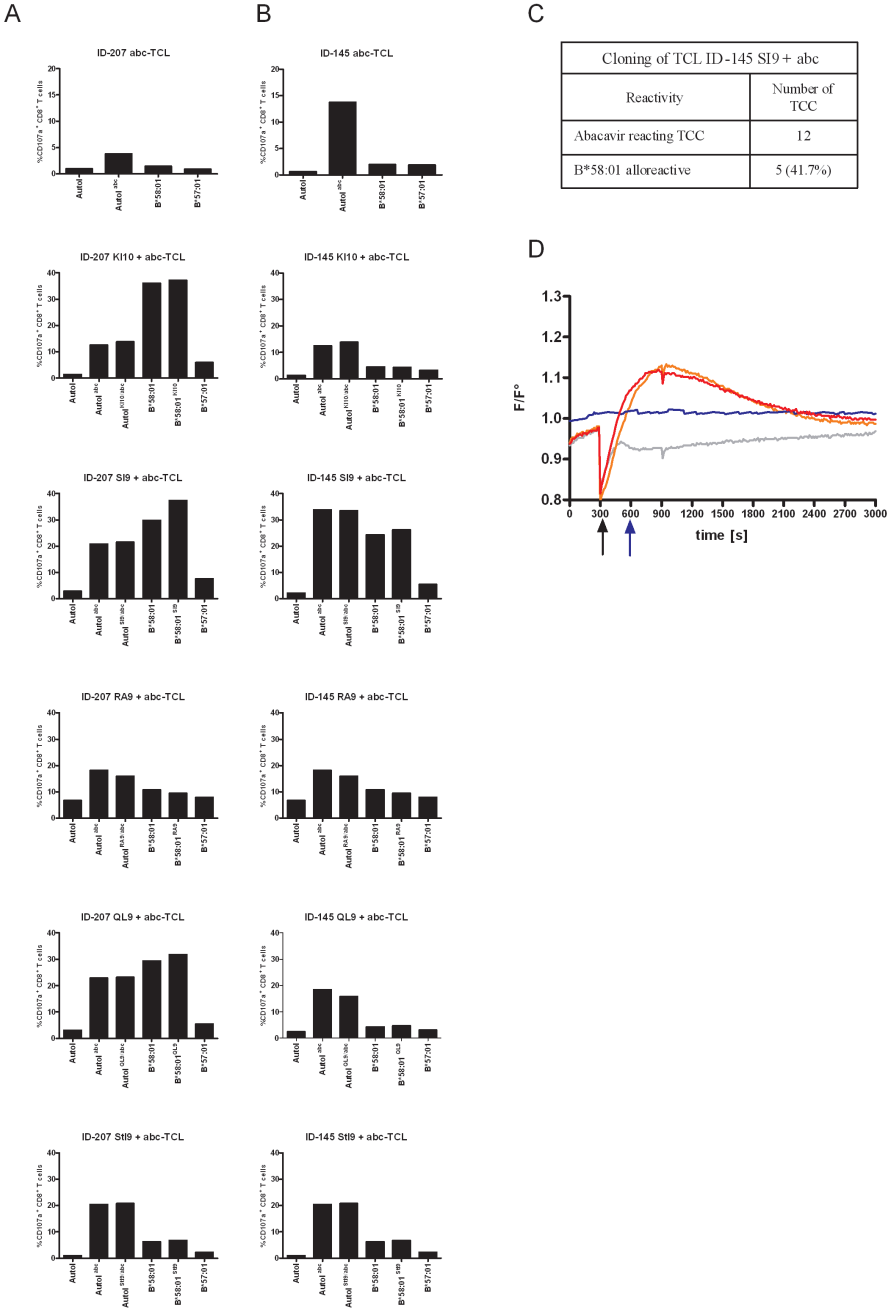
Stimulation with abacavir and selected peptides can enhance the allogenicity of the induced TCL. In order to generate TCL, PBMC from donors ID-207 (A) and ID-145 (B) were stimulated in the presence of abacavir (10 µg/ml) and the mentioned peptide (10 µg/ml). TCLs were re-challenged with autologous PHA-blasts (Autol.), autologous PHA-blasts pulsed with abacavir (Autol^abc^), or PHA-blasts pulsed with abacavir and the mentioned peptide (Autol^abc/xx9^), or 721.221 cells expressing HLA-B*58∶01 (B*58∶01), 721.221 cells expressing HLA-B*58∶01 and presenting the mentioned peptide (B*58∶01^XX9^), or 721.221 cells expressing HLA-B*57∶01 (B*57∶01). Reactivity was monitored by means of CD107a up-regulation on flow cytometry after 6 hours of stimulation. C. TCL SI9+ abc from ID-145 was cloned by limiting dilution. The table shows the number of generated TCC with reactivity to abacavir and cross-reactivity to HLA-B*58∶01. D. Calcium influx was measured by fluorescence measurement of the Fluo4-AM calcium sensitive dye. TCC B10 from ID-145 was stimulated after 300 seconds of baseline measurement (black arrow) with HLA-B*57∶01 expressing 721.211 cells (grey line), or HLA-B*57∶01 expressing 721.221 cells pulsed with abacavir (red line), or with 721.221 cells expressing HLA-B*58∶01 (orange line). Alternatively, after 600 seconds of baseline measurement (blue arrow) abacavir in solution (50 µg/ml) was added to the TCC in the presence of HLA-B*57∶01 expressing 721.221 cells (blue line). Representative data of two independent experiments are shown.

The solely allo-reactive allele HLA-B*58∶01 is very similar to HLA-B*57∶01 and differs in eight residues. Only one of them, Arg97 is located within the peptide binding groove at the anchoring site of the C-terminal residue. Three residues (Thr45, Glu46 and Leu103) are located on β-sheets below the peptide and four residues are located at a distance of the peptide interacting site. Of note, the top α-helices accessible to the TCR are identical on both molecules. Peptide anchoring residues for HLA-B*57∶01 and B*58∶01 are very similar, favouring a Trp or a Phe at the C-term of the presented peptide. However, some HLA-B*58∶01 restricted peptides have been observed with Ile or Val at the C-term [19], [20]. In recent studies, abacavir has been shown to modify the peptide repertoire binding onto the HLA-B*57∶01 molecule, favouring small aliphatic residues at the C-term instead of Trp of Phe [7]–[9]. We hypothesized that the observed allo-reactivity could be influenced by peptides with the following features: such peptides must show low binding capacity for HLA-B*57∶01, but high affinity for HLA-B*58∶01 as well as for {HLA-B*57∶01+abc}. To test this hypothesis, peptides eluted from {HLA-B*57∶01+abc} [8] but not found on HLA-B*57∶01 were analyzed for potential binding to HLA-B*58∶01. For that purpose, peptide affinities to the HLA-B*57∶01 and HLA-B*58∶01 molecules were calculated using the NetMHCpan server [12]. {HLA-B*57∶01+abc} binding peptides were thought to be possibly implied in the allo-reactivity if they showed strong to moderate affinity to HLA-B*58∶01 (IC_50_<1000 nM) and a low affinity for HLA-B*57∶01 in the absence of abacavir (IC_50_>5000 nM) (table 2). Peptides with the smallest IC_50_ ratio were considered as potential candidates affecting allo-reactivity. Peptides with a non-aliphatic residue at the C-term were excluded. Finally, five peptide candidates were selected (table 2), synthesized and added to PBMC of two HLA-B*57∶01^+^ individuals in the presence of abacavir. Subsequently, the generated TCL were assessed for abacavir reactivity and allo-reactivity to HLA-B*58∶01 (Fig. 6). TCL induced exclusively with abacavir did not show detectable allo-reactivity. In comparison, the addition of selected peptides during the induction phase increased the frequency of abacavir-reacting T cells (from 6.72±4.71% (abc) to 19.81±6.00% (abc+peptides), p = 0.013 by two-tailed Mann-Whitney test) and increased the allo-reactivity against HLA-B*58∶01 (from 1.70±0.38% (HLA-B*58∶01) to 16.26±12.27% (HLA-B*58∶01+ peptides), p = 0.041 by two-tailed Mann-Whitney test). In contrast the addition of the selected peptides during restimulation, rather than induction, did not enhance either the abacavir or allo-reactivity. Moreover, during the induction, the addition of peptides binding to HLA-B*57∶01 (and not to {HLA-B57∶01+abc}) did neither increase the fraction of abacavir-reacting T cells nor the HLA-B*58∶01 cross-reacting fraction (Fig. S4). From one TCL (HD-145, abc-SI9), TCC were generated by limiting dilution. Twelve abacavir-reacting TCC were selected, among which five (∼42%) were shown to be allo-reactive to HLA-B*58∶01 (Fig. 6 c).

### Allo-reactive TCC show a Delayed Activation

In a recent study, we observed at least two distinct patterns for abacavir reactivity. On the one hand, ‘low avidity’ TCC reacted only to APC previously incubated with the drug. On the other hand, ‘high avidity’ TCC reacted additionally to abacavir in solution, with an immediate activation kinetic [10]. To test the reactivity pattern of the allo-reactive TCC, calcium influx experiments were performed (Fig. 6d). Allo-reactive TCC were activated either by HLA-B*57∶01^+^ APC previously pulsed with abacavir or by HLA-B*58∶01^+^ APC. Abacavir added in solution was not able to induce calcium influx. Thus, we did not find any high avidity TCC reacting immediately to the drug, which were cross-reactive with the allo-antigen HLA-B*58∶01.

### Similar Surface Structure Model between HLA-B*58∶01 and {HLA-B*57∶01+abc} Presenting SI9 Peptide

**Figure 7:**
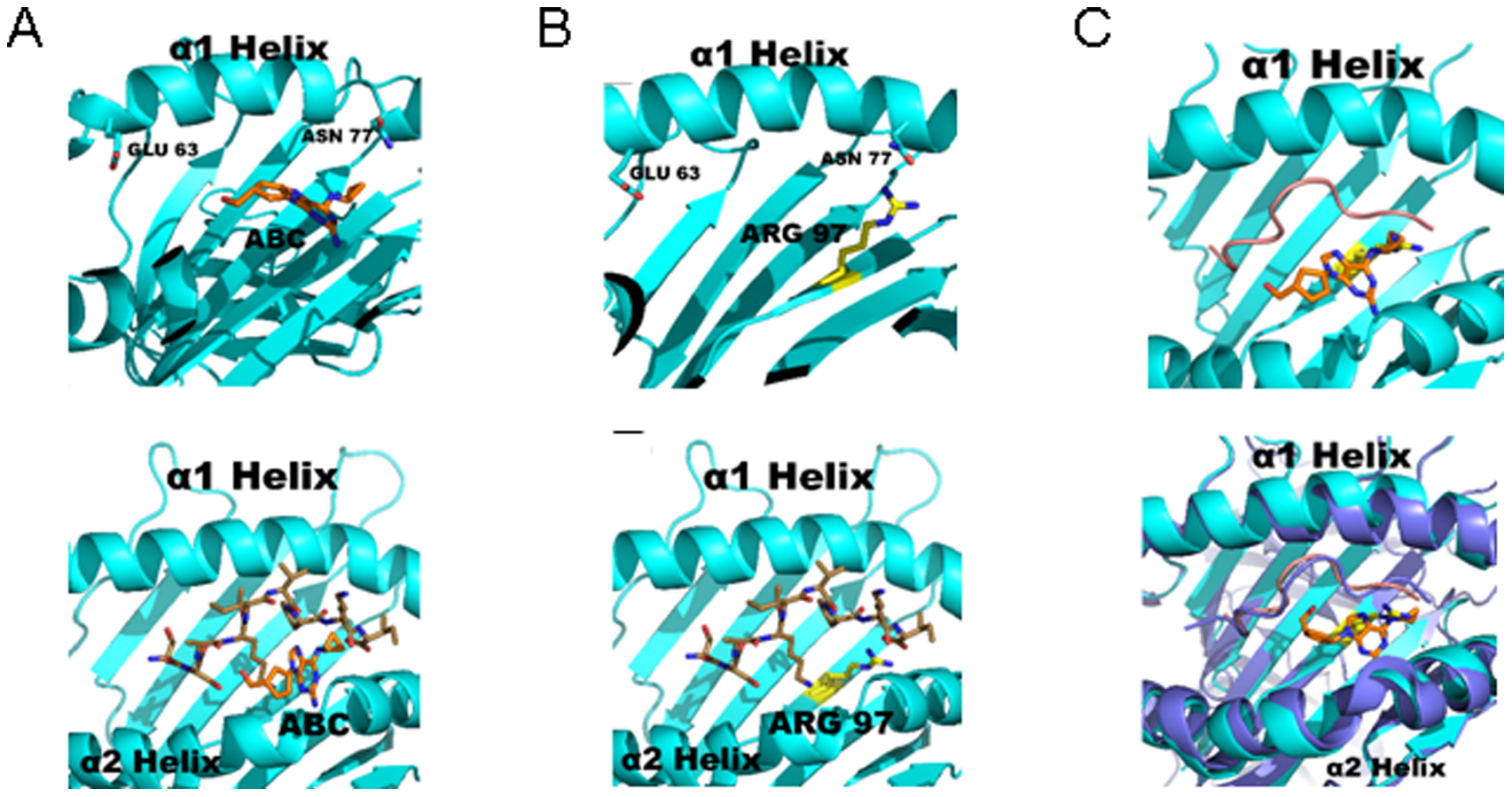
Molecular modelling of HLA-peptide complexes. A. HLA B*57∶01 peptide binding cleft with bound abacavir (orange) is shown empty (upper panel) or filled with the SI9 peptide (lower panel). B. The position of Arg97 is highlighted in yellow on the HLA-B*58∶01 molecule without (upper panel) or with the anchored SI9 peptide (lower panel). C An overlay of the {HLA-B*57∶01+abc} complex (turquoise) and HLA-B*58∶01 (purple) with modelled SI9 peptide is shown. SI9 highlighted in orange is bound to {HLA-B*57∶01+abc} and SI9 highlighted in purple is bound to HLA-B*58∶01. Abacavir and Arg97 found in the F9 pocket are highlighted in orange and yellow, respectively. The 3D conformation of SI9 is very similar between {HLA-B*57∶01+abc} and HLA-B*58∶01.

As the allo-reactivity towards HLA-B*58∶01 was enhanced by the addition of the SI9 peptide, we modelled the peptide onto the HLA-B*58∶01 molecule and onto {HLA-B*57∶01+abc}. The comparison of the F-pocket in HLA-B*58∶01 and {HLA-B*57∶01+abc} reveals a very similar 3D structure. Indeed, abacavir in complex with Val97 in {HLA-B*57∶01+abc} (Fig. 7a) shares partly the same location as Arg97 in HLA-B*58∶01 (Fig. 7 b). The static structures show that the three nitrogen-hydrogen groups of Arg97 can be overlapped with abacavir’s cyclopropane ring. These domains appear to co-ordinate the C-terminus of the anchored SI9. As the cyclopropane ring is very hydrophobic, it may build hydrophobic interactions with small aliphatic residues of the peptide C-terminus. The pyrimidine ring of abacavir does however not align with Arg97. Modelling peptide SI9 onto peptide binding grooves of {HLA-B*57∶01+abc} and HLA-B-*58∶01 revealed a very similar peptide conformation (Fig. 7a–c). Of note, the α-helices bordering the peptide groove are completely identical in HLA-B*57∶01 and HLA-B*58∶01. Based on these observations, we concluded that the structural surface accessible to the TCR was very similar if not identical in HLA-B*58∶01-SI9 complex and in the {HLA-B*57∶01+abc}-SI9 complex.

## Discussion

The association between HLA-B*57∶01 and abacavir hypersensitivity provides a unique opportunity to investigate the primary induction of drug-reacting T cells *in vitro*. In this report, primary induction of abacavir-reacting T cells occurred independently of co-stimulatory signals, since abacavir failed to induce DC maturation and T cell induction was successful in the absence of professional APC. Abacavir reactivity was found in cells from the naïve as well as the memory CD8^+^ T cell compartment. These features of abacavir-reacting T cells are shared with allo-reactive T cells. This allo-like feature of CD8 stimulation by {HLA-B*57∶01+abc+peptide} were confirmed by the finding that part of the abacavir-induced T cell clones were indeed allo-reactive to HLA-B*58∶01. This allo-reactivity was detected in 3/3 donors and was mainly induced by the peptide presented on {HLA-B*57∶01+abc}, as the addition of various peptides binding to {HLA-B*57∶01+abc} and HLA-B*58∶01 drastically increased the reactivity to abacavir and HLA-B*58∶01. Finally, *in silico* modelling of {HLA-B*57∶01+abc} or HLA-B*58∶01 in complex with the same peptide suggested an almost identical surface accessible to the TCR.

In agreement with previous observations and results, we were able to induce *in vitro* abacavir reactivity in HLA-B*57∶01^+^ donors after a 14 day culture. Overall, the induction of abacavir-reacting T cells in drug naïve individuals occurred faster than the generation of T cells for naïve peptides [21]. It was also faster than primary T cell inductions with carbamazepine [5], flucloxacillin [22] or allopurinol [23], requiring culture periods for 4–6 weeks to detect drug- reacting T cells. The ability to detect drug-reacting T cells in drug naïve individuals has implications for preclinical *in vitro* immuno-toxicological evaluations. It seems that rather long-lasting cell cultures and carefully selected read out systems are required to detect T cell reactivity to drugs. Proliferation assays, as done in memory CD4^+^ responses, are clearly insufficient to detect CD8^+^ T cell reactivity. Thereby, CD107a up-regulation was clearly more sensitive than IFNγ secretion in activated CD8^+^ T cells. Of note, these assays detect the risk for developing hypersensitivity, but are not suitable to discriminate between those patients who will develop abacavir-hypersensitivity clinically and abacavir-tolerant individuals. In other words, the risk allele and abacavir are sufficient to induce an immunological reactivity *in vitro*, but cannot predict the full clinical picture of abacavir hypersensitivity.

DC maturation is considered the essential first step to generate a primary immune response to novel antigens [24]. Nevertheless, the role of DC in initiating immune responses to drugs is unclear. Whereas DC maturation by haptens in contact hypersensitivity models has been well studied and confirmed [25], the source and the role of co-stimulation for other mechanisms involved in drug HR have been questioned. Without hapten formation, immune activation by drugs might bypass the innate immune system [26]. The data obtained in this study with abacavir support this assumption. We did not find any evidence for abacavir-induced DC activation. Neither up-regulation of rather sensitive maturation markers nor secretion of pro-inflammatory cytokines was detected upon abacavir exposure. Since it is difficult to conclude from a missing signal to its absence, these findings were confirmed by the successful induction of abacavir-reacting T cells in the absence of monocytes or DC in the culture. Thus, our data argue against a need for DC activation in the primary response to abacavir.

The fact that co-stimulatory signals are not required for the induction of abacavir reactivity suggests that abacavir-reacting T cells originate from the memory compartment. On second antigen exposure, memory T cells do not require further co-stimulation. For this reason we already hypothesized in previous reports, that p-i reacting T cells stemmed from the memory pool [6]. However, this study demonstrates that both pools, naïve and memory, reacted to abacavir. We think that this observation should be confirmed in other ‘p-i HLA’ driven HR. The characteristics of abacavir-reacting T cells are shared by allo-reacting T cells. We had already reported that a high proportion (27%) of CD4^+^ T cells specific for lidocaine or sulfamethoxazole were allo-reactive [27]. To our knowledge, this is the first time allo-reactivity of MHC-class I restricted drug-reacting CD8^+^ T cells is described.

In this study, only HLA-B*58∶01 was shown to be allo-stimulatory. Since only 5% of TCC were allo-reactive, allo-reactivity could be detected at the TCC level but not at the TCL level.

Nevertheless, it was not a random phenomenon, as this allo-reactivity was consistently found in all included donors (3/3) with similar frequencies (table 3). We believe that the above described features of allo-reactivity apply to *all* {HLA-B*57∶01+abc}-reacting T cells, and not only for those TCC with HLA-B*58∶01-crossreactivity. Additional cross-reactivities to other allo-HLA might exist as well, probably with closely related subtypes, like HLA-B*57∶02 or HLA-B*57∶03. Screening for allo-reactivity with a broad panel of transfectant cells expressing closely related alleles might clarify this issue.

Allo-reactivity between closely related alleles has already been reported for anti-viral responses [28]. HLA-B*57∶01 and HLA-B*58∶01 molecules share very similar 3D structures as well as similarities in the repertoire of presented peptides. This raises the possibility that the cross-reactivity of abacavir-induced TCC may be due to the presentation of similar/identical peptides. Actually, two different approaches – addition of peptides in abacavir induction cultures and molecular modeling - underlined the important role of peptides in abacavir induced T cell reactivity. The addition of extracellular peptides increased not only the proportion of allo-reactive T cells, but also the frequency of abacavir-reacting cells. Therefore, we assume that {HLA-B*57∶01+abc} was stabilized by the extracellular peptides, increasing the immunogenicity of the entire complex. Moreover, we think that ‘peptide bulging’ above Arg97 or above Val97+ abacavir in HLA-B*58∶01 and {HLA-B*57∶01+abc}, respectively, may provide the allo-signal for the involved TCR. Thus, abacavir-induced reactivity is peptide dependent. This is in agreement with the observed delayed activation kinetic of allo-reacting TCC.

However, an exclusive peptide specificity of abacavir-reacting TCC could not be demonstrated. First, the use of various APC (DC, EBV-BLCL, activated T cells and HLA-B*57∶01 transfected cell lines) all leaded to strong TCC/TCL activation. Secondly, in a previous study a substantial portion of abacavir-reacting TCC was immediately activated upon abacavir supply [10]. This activation was too fast to allow altered peptide loading onto HLA molecules within the ER. Therefore, it is difficult to attribute the specificity of such TCC to a single peptide.

A possible explanation for the ‘peptide dependence but not peptide specificity’ of these T cells may lie in the poly-specificity of allo-reactive T cells. Allo-specific TCR can recognize different unrelated peptides presented on the same allo-HLA molecule [16]. The same polyspecificity might occur, if the peptides are recognized on the background of an ‘altered self-allele’ like {HLA-B*57∶01+abc}. The modification of the F-pocket by abacavir might be the common denominator for the recognition of various peptides. Moreover, this polyspecificity of allo-specific T cells is generally thought to explain the strength of allo-responses [18], and may also explain the strong abacavir reactivity. In the present pi-HLA situation, abacavir can bind and alter HLA-B*57∶01 molecule either within the ER, thereby promoting the loading of altered peptides, or directly and fast on the cell surface, modifying the structure of already anchored peptides.

In conclusion, we show that *in vitro* generated abacavir-reacting T cells follow the rules of an allo-immune response. Abacavir-induced T cells do not require co-stimulatory signals and arise from the naïve and memory T cell pools. The allo-reactivity is exemplified for the allele HLA-B*58∶01 and was enhanced by adding HLA-B*58∶01 binding peptides to T cell cultures during the abacavir induction phase. Whether the abacavir induced ‘altered self-allele’ is exceptional or can be applied to other drug HR or environmental toxicities is still unclear and will need further investigations. Besides, clinical observations [29], [30] on graft versus host disease have shown similarities with toxic epidermal necrolysis. These observations together with our study support a common mechanism for the pathophysiology of both diseases.

## Supporting Information

Figure S1 **Comparison between degranulation (CD107a) and IFNγ secretion.** PBMC from donor ID-585 were stimulated with abacavir for 14 days (A) or 28 days (B). Cells were re-challenged in the presence of autologous PBMC with (lower plots) or without (upper plots) abacavir (10 mg/ml) for four hours. CD107a up-regulation and IFNy secretion was analyzed by flow cytometry. Percentages indicate the fractions of the positive cell populations within the CD3+ CD8+ T cell gate, expressing CD107a only or CD107a and IFNγ.
(PDF)

Figure S2 **Naïve and memory sorting efficiency.** PBMC from ID-576 were stained for naive (CD45RA, CCR7; left plots) and memory (CD45RO; right plots) markers, before (A) and after magnetic sorting for memory (B) and naive (C) CD8+ T cell enrichment. Cells were gated on CD3+ CD8+ events (left plots). Percentages indicate the cell fractions within the corresponding squares.
(PDF)

Figure S3 **Allo-reactive TCC are stimulated by different types of APC.** TCC B10 from ID-145 was stimulated with. 221 cells expressing HLA-B*57∶01. 221 cells expressing HLA-B*57∶01 pulsed with abacavir (10 µg/ml). 221 cells expressing HLA-B*58∶01, PHA blasts from donor ID-601 (HLA-B*58∶01+) and PBMC from donor ID-601 (HLA-B*58∶01+). All these APC were previously stained with CFSE and then excluded from the analyzed CD8+ T cell gate. After a four hours re-challenge, cells were analyzed by flow cytometry. Plots are gated on CD3+, CFSE- cells and percentages of CD8+ CD107a+ T cells are indicated above each plot.
(PDF)

Figure S4 No increase of cross-allo-reactivity after abacavir priming in the presence of peptides binding to HLA-B*57∶01. PBMC from donors HD-685 (A) and HD-630 (B) were cultured in the presence of abacavir (10 ug/ml) with either KF11 peptide (KAFSPEVIPMF) or IsW9 (ISPRTLNAW) (10 ug/ml). Both peptides derive from HIVgag protein. After two weeks of *in vitro* induction, cells were re-challenged with 722.221 cells expressing HLA-B*57∶01 (.221 B*57∶01), or 722.221 cells expressing B*57∶01 in the presence of abacavir (.221 B*57∶01+ abacavir) or 722.221 cells transduced with HLA-B*58∶01 (.221 B*58∶01). Degranulation was measured after four hours of re-stimulation, by CD107a staining on FACS. Results were gated on CD3+, CD8+ cells.
(PDF)
