# Supplementary material for: Abacavir Induced T Cell Reactivity from Drug Naïve Individuals Shares Features of Allo-Immune Responses
Source: PLoS One. 2014 Apr 21;9(4):e95339. doi: 10.1371/journal.pone.0095339 (PMC3994040; doi:10.1371/journal.pone.0095339)
Supplement: Figure S1 — Comparison between degranulation (CD107a) and IFNγ secretion. PBMC from donor ID-585 were stimulated with abacavir for 14 days (A) or 28 days (B). Cells were re-challenged in the presence of autologous PBMC with (lower plots) or without (upper plots) abacavir (10 mg/ml) for four hours. CD107a up-regulation and IFNy secretion was analyzed by flow cytometry. Percentages indicate the fractions of the positive cell populations within the CD3+ CD8+ T cell gate, expressing CD107a only or CD107a and IFNγ. (PDF) [file pone.0095339.s001.pdf]

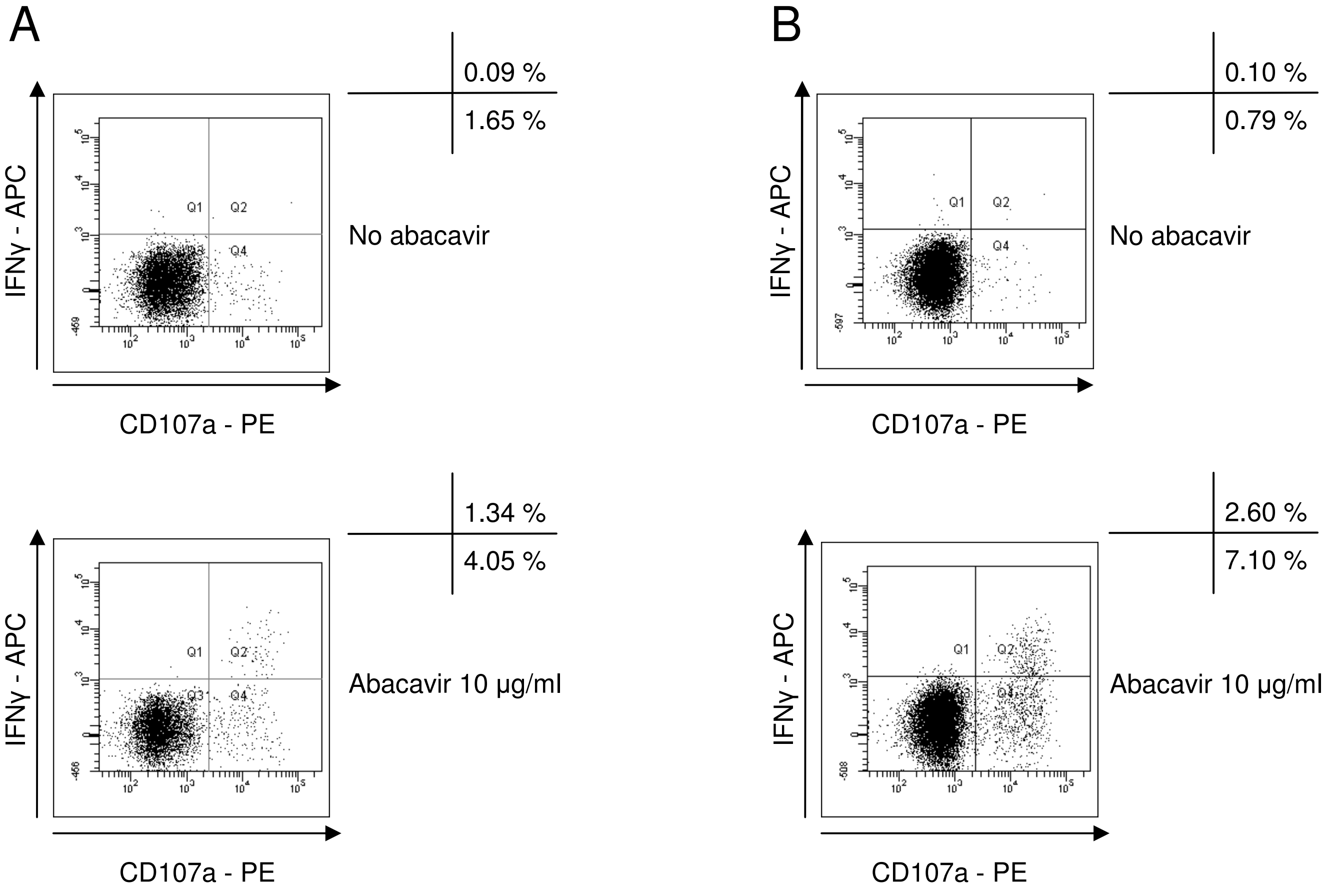

Suppl. Figure S1. Comparison between degranulation (CD107a) and IFNγ secretion.

PBMC from donor ID-585 were stimulated with abacavir for 14 days (A) or 28 days (B). Cells were re-challenged in the presence of autologous PBMC with (lower plots) or without (upper plots) abacavir (10µg/ml) for four hours. CD107a up-regulation and IFNγ secretion was analyzed by flow cytometry. Percentages indicate the fractions of the positive cell populations within the $CD3^+$ $CD8^+$ T cell gate, expressing CD107a only or CD107a and IFNγ.
