# Supplementary material for: Abacavir Induced T Cell Reactivity from Drug Naïve Individuals Shares Features of Allo-Immune Responses
Source: PLoS One. 2014 Apr 21;9(4):e95339. doi: 10.1371/journal.pone.0095339 (PMC3994040; doi:10.1371/journal.pone.0095339)
Supplement: Figure S2 — Naïve and memory sorting efficiency. PBMC from ID-576 were stained for naive (CD45RA, CCR7; left plots) and memory (CD45RO; right plots) markers, before (A) and after magnetic sorting for memory (B) and naive (C) CD8+ T cell enrichment. Cells were gated on CD3+ CD8+ events (left plots). Percentages indicate the cell fractions within the corresponding squares. (PDF) [file pone.0095339.s002.pdf]

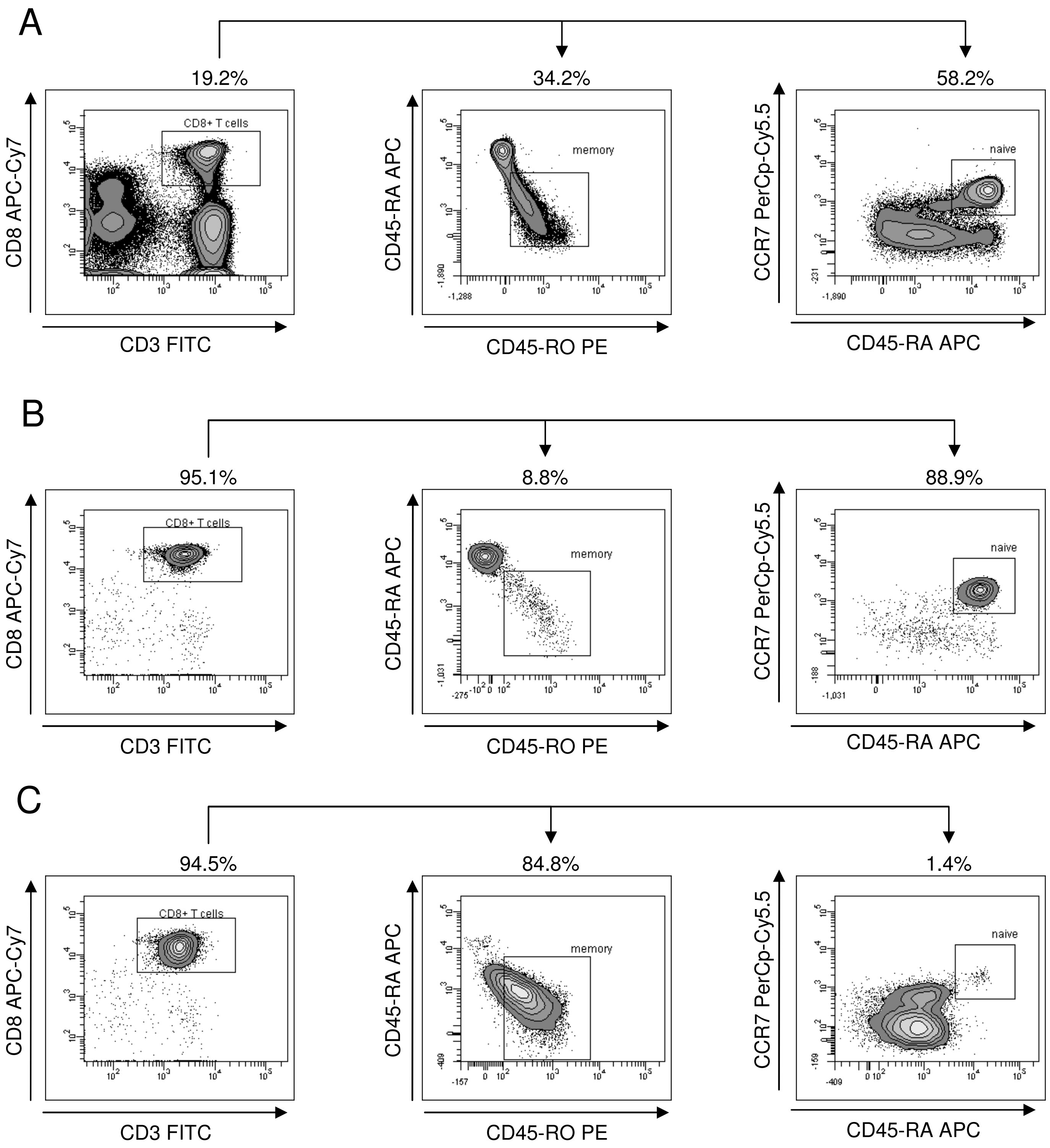

Suppl. Figure S2. Naïve and memory sorting efficiency

PBMC from ID-576 were stained for naive (CD45RA, CCR7; left plots) and memory (CD45RO; right plots) markers, before (A) and after magnetic sorting for memory (B) and naive (C) $CD8^+$ T cell enrichment . Cells were gated on $CD3^+$ $CD8^+$ events (left plots). Percentages indicate the cell fractions within the corresponding squares.
