# Supplementary material for: Abacavir Induced T Cell Reactivity from Drug Naïve Individuals Shares Features of Allo-Immune Responses
Source: PLoS One. 2014 Apr 21;9(4):e95339. doi: 10.1371/journal.pone.0095339 (PMC3994040; doi:10.1371/journal.pone.0095339)
Supplement: Figure S3 — Allo-reactive TCC are stimulated by different types of APC. TCC B10 from ID-145 was stimulated with. 221 cells expressing HLA-B*57∶01. 221 cells expressing HLA-B*57∶01 pulsed with abacavir (10 µg/ml). 221 cells expressing HLA-B*58∶01, PHA blasts from donor ID-601 (HLA-B*58∶01+) and PBMC from donor ID-601 (HLA-B*58∶01+). All these APC were previously stained with CFSE and then excluded from the analyzed CD8+ T cell gate. After a four hours re-challenge, cells were analyzed by flow cytometry. Plots are gated on CD3+, CFSE- cells and percentages of CD8+ CD107a+ T cells are indicated above each plot. (PDF) [file pone.0095339.s003.pdf]

TCC ID-145 B10

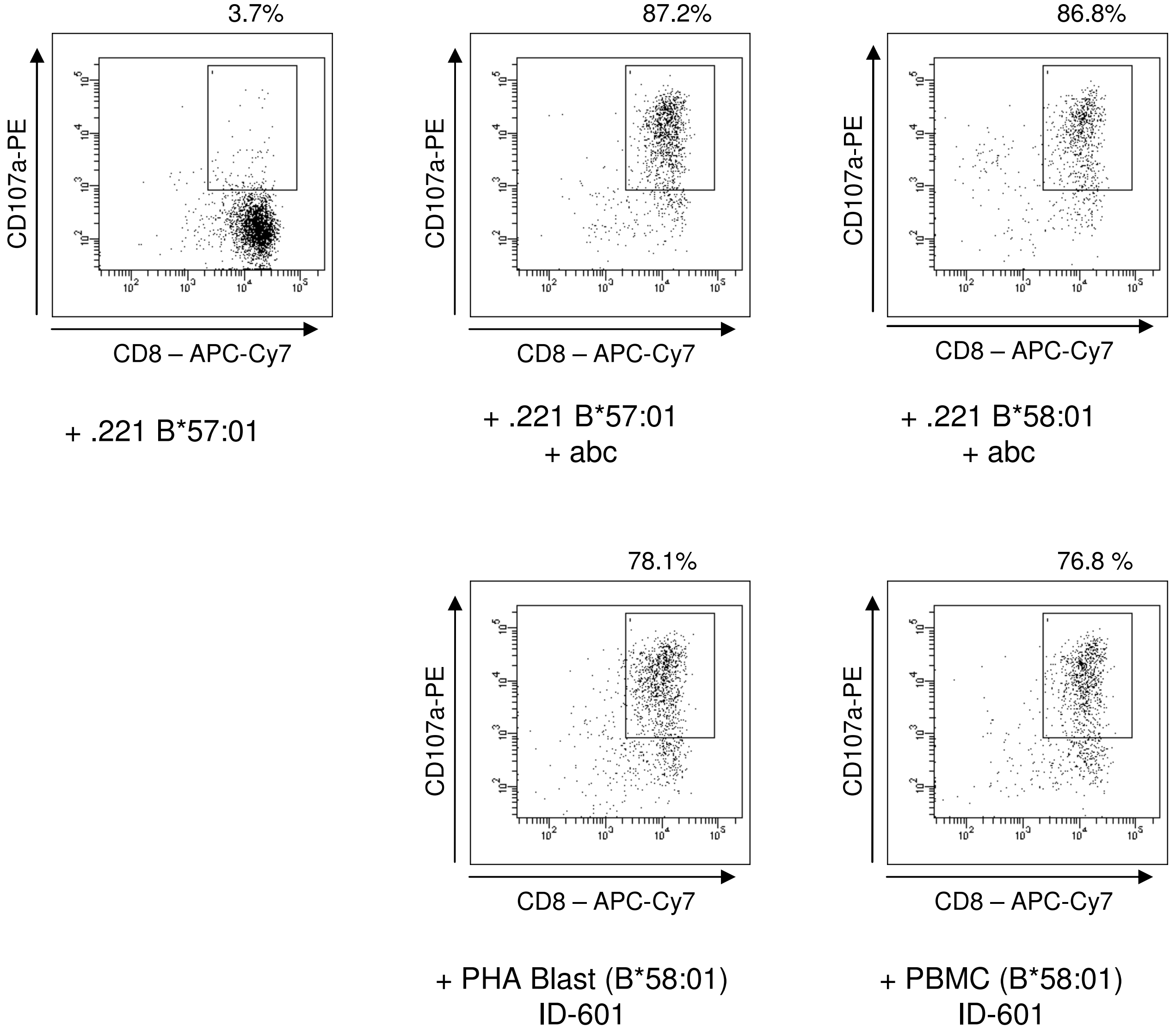

Suppl. Figure S3. Allo-reactive TCC are stimulated by different types of APC.
TCC B10 from ID-145 was stimulated with .221 cells expressing HLA-B*57:01, .221 cells expressing HLA-B*57:01 pulsed with abacavir (10 µg/ml), .221 cells expressing HLA-B*58:01, PHA blasts from donor ID-601 (HLA-B*58:01^{+}) and PBMC from donor ID-601 (HLA-B*58:01^{+}). All these APC were previously stained with CFSE and then excluded from the analyzed CD8^{+} T cell gate. After a four hours re-challenge, cells were analyzed by flow cytometry. Plots are gated on CD3$^{+,}$ CFSE^{-} cells and percentages of CD8^{+} CD107a^{+} T cells are indicated above each plot.
