# Supplementary material for: Abacavir Induced T Cell Reactivity from Drug Naïve Individuals Shares Features of Allo-Immune Responses
Source: PLoS One. 2014 Apr 21;9(4):e95339. doi: 10.1371/journal.pone.0095339 (PMC3994040; doi:10.1371/journal.pone.0095339)
Supplement: Figure S4 — No increase of cross-allo-reactivity after abacavir priming in the presence of peptides binding to HLA-B*57∶01. PBMC from donors HD-685 (A) and HD-630 (B) were cultured in the presence of abacavir (10 ug/ml) with either KF11 peptide (KAFSPEVIPMF) or IsW9 (ISPRTLNAW) (10 ug/ml). Both peptides derive from HIVgag protein. After two weeks of in vitro induction, cells were re-challenged with 722.221 cells expressing HLA-B*57∶01 (.221 B*57∶01), or 722.221 cells expressing B*57∶01 in the presence of abacavir (.221 B*57∶01+ abacavir) or 722.221 cells transduced with HLA-B*58∶01 (.221 B*58∶01). Degranulation was measured after four hours of re-stimulation, by CD107a staining on FACS. Results were gated on CD3+, CD8+ cells. (PDF) [file pone.0095339.s004.pdf]

A

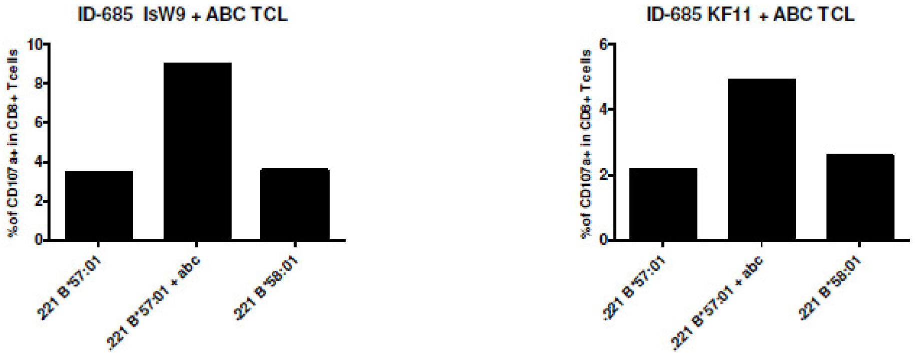

B

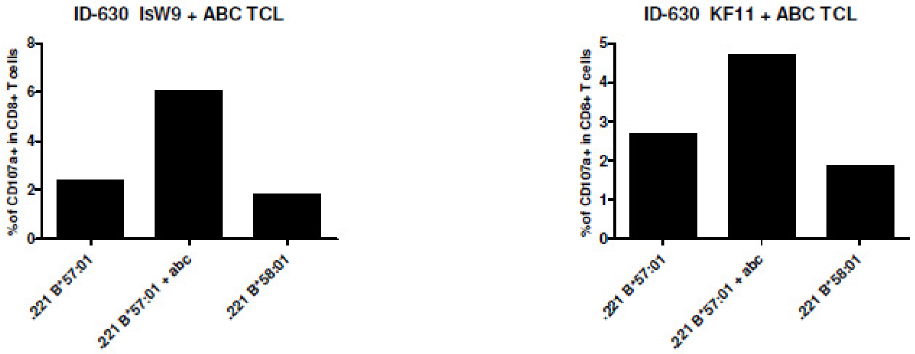

Suppl. Figure S4. No increase of cross-allo-reactivity after abacavir priming in the presence of peptides binding to HLA-B*57:01

PBMC from donors HD-685 (A) and HD-630 (B) were cultured in the presence of abacavir (10ug/ml) with either KF11 peptide (KAFSPEVIPMF) or IsW9 (ISPRTLNAW) (10ug/ml). Both peptides derive from HIVgag protein. After two weeks of *in vitro* induction, cells were re-challenged with 722.221 cells expressing HLA-B*57:01 (.221 B*57:01), or 722.221 cells expressing B*57:01 in the presence of abacavir (.221 B*57:01 + abacavir) or 722.221 cells transduced with HLA-B*58:01 (.221 B*58:01). Degranulation was measured after four hours of re-stimulation, by CD107a staining on FACS. Results were gated on $CD3^+$, $CD8^+$ cells.
